# Microwave-Assisted Extraction of Natural Antioxidants from the Exotic *Gordonia axillaris* Fruit: Optimization and Identification of Phenolic Compounds

**DOI:** 10.3390/molecules22091481

**Published:** 2017-09-06

**Authors:** Ya Li, Sha Li, Sheng-Jun Lin, Jiao-Jiao Zhang, Cai-Ning Zhao, Hua-Bin Li

**Affiliations:** 1 Guangdong Provincial Key Laboratory of Food, Nutrition and Health, Department of Nutrition, School of Public Health, Sun Yat-Sen University, Guangzhou 510080, China; liya28@mail2.sysu.edu.cn (Y.L.); zhangjj46@mail2.sysu.edu.cn (J.-J.Z.); zhaocn@mail2.sysu.edu.cn (C.-N.Z.); 2 School of Chinese Medicine, Li Ka Shing Faculty of Medicine, The University of Hong Kong, Hong Kong 999077, China; 3 Zhongshan Center for Disease Control and Prevention, Zhongshan 528403, China; zscdclsj@163.com; 4 South China Sea Bioresource Exploitation and Utilization Collaborative Innovation Center, Sun Yat-Sen University, Guangzhou 510006, China

**Keywords:** *Gordonia axillaris*, fruit, microwave-assisted extraction, response surface methodology, antioxidant, phenolic compounds

## Abstract

Our previous study reported that the fruit of *Gordonia axillaris*, an edible wild fruit, possessed strong antioxidant activity. In this study, a microwave-assisted extraction (MAE) method was established to extract antioxidants from the fruit of *Gordonia axillaris*. The influence of five parameters, including ethanol concentration, solvent/material ratio, extraction time, extraction temperature and microwave power, was investigated by single-factor experiments. Three factors, namely ethanol concentration, solvent/material ratio, extraction time, were found to exert a major influence on extraction efficacy, and were further studied by response surface methodology to investigate their interactions. Ethanol concentration of 36.89%, solvent/material ratio of 29.56 mL/g, extraction time of 71.04 min, temperature of 40 °C, and microwave power of 400 W were found to be the optimal condition. The TEAC value was 198.16 ± 5.47 µmol Trolox/g DW under the optimal conditions, which was in conformity to the predicted value (200.28 µmol Trolox/g DW). In addition, the MAE method was compared with two conventional methods (Soxhlet extraction and maceration extraction). Results showed that the antioxidant capacity of the extract obtained by MAE method was stronger than that obtained by maceration (168.67 ± 3.88 µmol Trolox/g DW) or Soxhlet extraction (114.09 ± 2.01 µmol Trolox/g DW). Finally, several phenolic compounds in the extract were identified and quantified by UPLC-MS/MS, which were rutin, gallic acid, protocatechuic acid, epicatechin, epicatechin gallate, 2-hydrocinnamic acid, *p*-coumaric acid, quercetin, chlorogenic acid and ferulic acid.

## 1. Introduction

In recent years, free radicals, including reactive oxygen species (ROS) and reactive nitrogen species (RNS), have gained increasing attention [1]. Free radicals are produced during normal cellular metabolism, and the balanced system of free radicals and antioxidants in human allows some free radicals to perform useful functions [2]. However, excessive free radicals could induce oxidative damage, which may partly contribute to various human diseases, such as cancer and neurodegenerative disease [3,4]. Many natural products, such as fruits, vegetables and edible flowers, have been found to be rich in antioxidants and thus exhibit great antioxidant capacity in vitro [5,6,7,8,9,10,11,12,13]. Therefore, studies on the extraction and identification of these antioxidants are of great value. 

**Figure 1:**
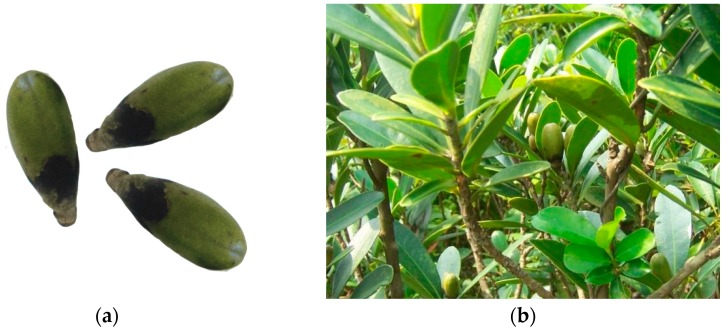
The fruit (**a**) and leaves (**b**) of *Gordonia axillaris*.

*Gordonia axillaris* (Figure 1) belongs to the Theaceae family [14]. In China, it is widely distributed in southern regions such as Hong Kong, Taiwan and Hainan. The tree itself is usually planted as a garden ornamental due to its evergreen character and attractive appearance, and the leaf and fruit of *Gordonia axillaris* have also been used in Chinese folk medicine to treat some ailments such as stomach aches and diarrhea [15,16]. Wang et al. have reported that camelliin B, a tannin isolated from the leaves of *Gordonia axillaris*, showed selective cytotoxic effects in a HeLa cervical carcinoma cell line [17]. In our previous study, it was found that the fruit of *Gordonia axillaris* exhibited strong in vitro antioxidant activity [5]. Therefore, the fruit of *Gordonia axillaris* is a potentially valuable but underutilized resource and demands further investigation. In order to study chemical composition and bioactivities of the fruit of *Gordonia axillaris*, extraction of bioactive components is a critical step.

Various extraction methods have been developed to extract bioactive components from natural products, including some innovative technologies such as microwave-assisted extraction (MAE), ultrasound-assisted extraction, enzyme-assisted extraction, and some conventional methods such as maceration and Soxhlet extraction [18,19,20,21]. Among these methods, MAE is a green and effective extraction method. Compared with conventional extraction methods, MAE has some advantages, such as shorter extraction time and lower temperature, which leads to less degradation of thermally labile compounds [22,23,24]. Besides, it has also been found that MAE is compatible with water as an extraction solvent so that the use of organic solvents is reduced/eliminated, and more medium- and non-polar organic compounds from plant materials could also be extracted by MAE [25,26,27]. Moreover, MAE as a sample preparation method could be coupled with some chromatography and spectroscopy tools, such as HPLC and GC-MS, to achieve rapid determination with satisfactory precision and recovery [28,29,30]. Therefore, MAE has been used in the extraction of several bioactive components from natural products [31,32].

During the MAE process, several factors could influence the extraction efficacy, such as the extraction time and power, temperature, the solvent composition and solvent/material ratio [33]. These factors not only directly influence the extraction efficacy, but also interact with each other [34,35,36,37,38]. Therefore, the interaction effects of these factors need to be evaluated. The response surface methodology (RSM) has been recently used for the modeling and optimization of extraction conditions [39]. In the present study, a microwave-assisted extraction method was established to extract natural antioxidants from the fruit of *Gordonia axillaris*. Single-factor experiments were carried out to study the effect of individual parameters on extraction efficacy, and RSM was then utilized to investigate the interactions between the major influential parameters. In addition, maceration and Soxhlet extraction were conducted to compare the respective efficacy. Finally, the antioxidant components in the extract were analyzed by UPLC-MS/MS.

## 2. Results and Discussion

### 2.1. Single-Factor Experiments

#### 2.1.1. Effect of Ethanol Concentration

**Figure 2:**
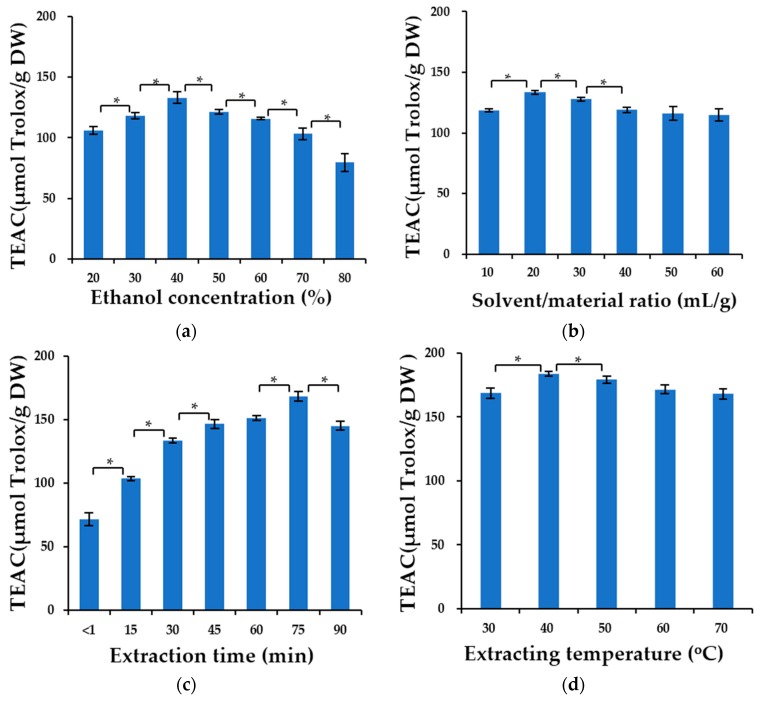

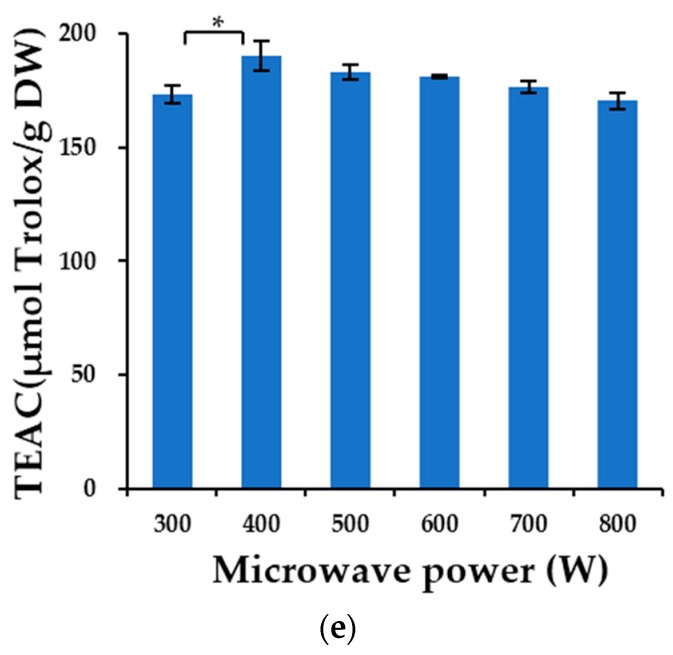
Effects of ethanol concentration (**a**); solvent/material ratio (**b**); extraction time (**c**); extraction temperature (**d**); and microwave power (**e**) on the extraction efficacy. Note: * Significant difference (*p* < 0.05).

In this study, aqueous ethanol solution was chosen as extraction solvent due to its low toxicity and the easy accessibility of water and ethanol, which have been widely used in the extraction of natural antioxidants [40,41]. In this section, the effect of different ethanol concentrations (20%, 30%, 40%, 50%, 60%, 70% and 80%) on the extraction efficacy was investigated under the following conditions: solvent/material ratio of 20 mL/g, extraction time of 30 min, extraction temperature of 30 °C, and microwave power of 500 W. Figure 2a shows that as the ethanol concentration increased from 20% to 40%, the Trolox equivalent antioxidant capacity (TEAC) values increased from 105.98 ± 3.08 to 133.16 ± 4.68 µmol Trolox/g DW. However, when the ethanol concentration continued to increase from 40% to 80%, a decreasing trend was observed in the TEAC value. Therefore, an ethanol concentration of 40% was chosen in the subsequent experiments.

#### 2.1.2. Effect of Solvent/Material Ratio

The solvent/material ratio (S/M ratio) could affect the extraction efficacy because within certain limits a higher ratio could cause a higher concentration difference, which is beneficial for the mass transfer and dissolution of solutes [37]. The effect of S/M ratio on extraction efficacy was investigated in the range of 10–60 mL/g, while the other factors were controlled as follows: ethanol concentration of 40%, extraction time of 30 min, extraction temperature of 30 °C, and microwave power of 500 W.

As shown in Figure 2b, the TEAC value increased significantly as the S/M ratio increased from 10 to 20 mL/g, and then gradually decreased as the S/M ratio was increased from 20 to 50 mL/g, and finally remained almost unchanged for S/M ratios from 50–60 mL/g. A possible reason is that when the S/M ratio reached 20 mL/g, the mass transfer process reached its maximum. Similar results were reported in the studies on extraction of polysaccharides from *Gentiana scabra* [42] and extraction of flavonoids from *Crotalaria sessiliflora* [29]. Thus, a S/M ratio of 20 mL/g was selected in the subsequent experiments.

#### 2.1.3. Effect of Extraction Time

The effect of different extraction times (<1, 15, 30, 45, 60, 75 and 90 min) on the extraction efficacy was investigated under the following conditions: ethanol concentration of 40%, S/M ratio of 20 mL/g, extraction temperature of 30 °C and microwave power of 500 W. As seen from Figure 2c, the TEAC value increased from 71.72 ± 5.15 to 168.46 ± 3.54 µmol Trolox/g DW as the extraction time was increased from 0 to 75 min. When the extraction time continued to increase, the extraction efficacy began to fall. A possible reason is that a long microwave exposure might cause the degradation of some antioxidants. Similar results were reported in studies on the extraction of anthocyanins from grape juice waste [43] and polysaccharides from *Moringa oleifera* Lam. leaves [44]. Thus, an extraction time of 75 min was selected in the subsequent experiments.

#### 2.1.4. Effect of Extraction Temperature

The effect of different extraction temperatures (30, 40, 50, 60, and 70 °C) was investigated. The other factors were controlled as follows: ethanol concentration of 40%, S/M ratio of 20 mL/g, extraction time of 75 min and microwave power of 500 W (Figure 2d). When the extraction temperature increased from 30 to 40 °C, the TEAC value increased from 168.46 ± 4.05 to 183.51 ± 1.80 µmol Trolox/g DW. Then a downward trend in extraction efficacy was observed as the extraction temperature was further increased from 40 to 70 °C. This phenomenon might be attributed to the fact that higher temperatures (>40 °C) caused decomposition of some thermolabile antioxidants [45]. Therefore, the extraction temperature of 40 °C was used in the subsequent experiments.

#### 2.1.5. Effect of Microwave Power

The microwave power could also influence the yield of antioxidants in the MAE process. Figure 2e shows the impact of different microwave power settings (300, 400, 500, 600, 700 and 800 W) on the extraction efficacy under the conditions of ethanol concentration of 40%, S/M ratio of 20 mL/g, extraction time of 75 min and extraction temperature of 40 °C. According to the results, the TEAC value increased with the microwave power increasing from 300 to 400 W, and reached the peak (190.33 ± 6.57) at microwave power of 400 W. As the microwave power continued to increase, the TEAC value dropped gradually. A possible reason is that during the MAE process, the microwave power has a dual influence on the extraction efficacy. On the one hand, an increase in microwave power could accelerate the solvent’s movement, cell rupture and diffusion of extractives into the solvent, thereby increasing the extraction efficacy. On the other hand, excessive microwave power (>400 W in this study) could cause the degradation of some antioxidants [46,47]. Hence, a microwave power of 400 W was selected as the optimal microwave power in the experiments. 

### 2.2. Results of Response Surface Methodology Experiments

#### 2.2.1. Central Composite Rotatable Design (CCRD) and Results

Based on the results of single-factor experiments, three factors (ethanol concentration, S/M ratio and extraction time) were seen to exert a greater influence on the extraction efficacy than the other two. Thus, they were chosen in the RSM design for further optimization. The middle level was set as 40% ethanol, 20 mL/g S/M ratio, and 75 min extraction time. The extraction temperature and microwave power were fixed at 40 °C and 400 W, respectively. The experimental design of 20 runs and the corresponding response values as well as the predicted values are displayed in Table 1. The results showed that the antioxidant capacity varied from 43.27 to 196.88 µmol Trolox/g DW.

#### 2.2.2. Fitting the Model 

In this part, multiple regression fitting was used to analyze the data from Table 1, and a quadratic polynomial regression model equation (Equation (1)) was generated, which described the relationship between the TEAC value (Y) and the three variables:

Y = 179.29 − 8.75X_1_ + 39.15X_2_ − 6.97X_3_ + 0.77X_1_X_2_ + 3.37X_1_X_3_ + 0.87X_2_X_3_ − 14.28X_1_^2^ − 20.24X_2_^2^ − 13.64X_3_^2^ (1)

Analysis of variance (ANOVA) was carried out to evaluate the validity of the fitted model as displayed in Table 2. The model *F*-value of 12.49 and low *p* value of 0.0002 (<0.05) implies the generated model is significant. The determination coefficient value (R^2^) of 0.9183, and the adjusted R^2^ value (Adj. R^2^) of 0.8448 indicated a satisfactory correlation between responses and independent variables. In addition, the validity of the model was confirmed using lack of fit testing. The low *F* value (*F* = 0.67) and high *p* value (*p* = 0.6663) of “lack of fit” suggested the suitability of this model to accurately predict the variation [48].

#### 2.2.3. Response Surfaces Analysis

**Figure 3:**
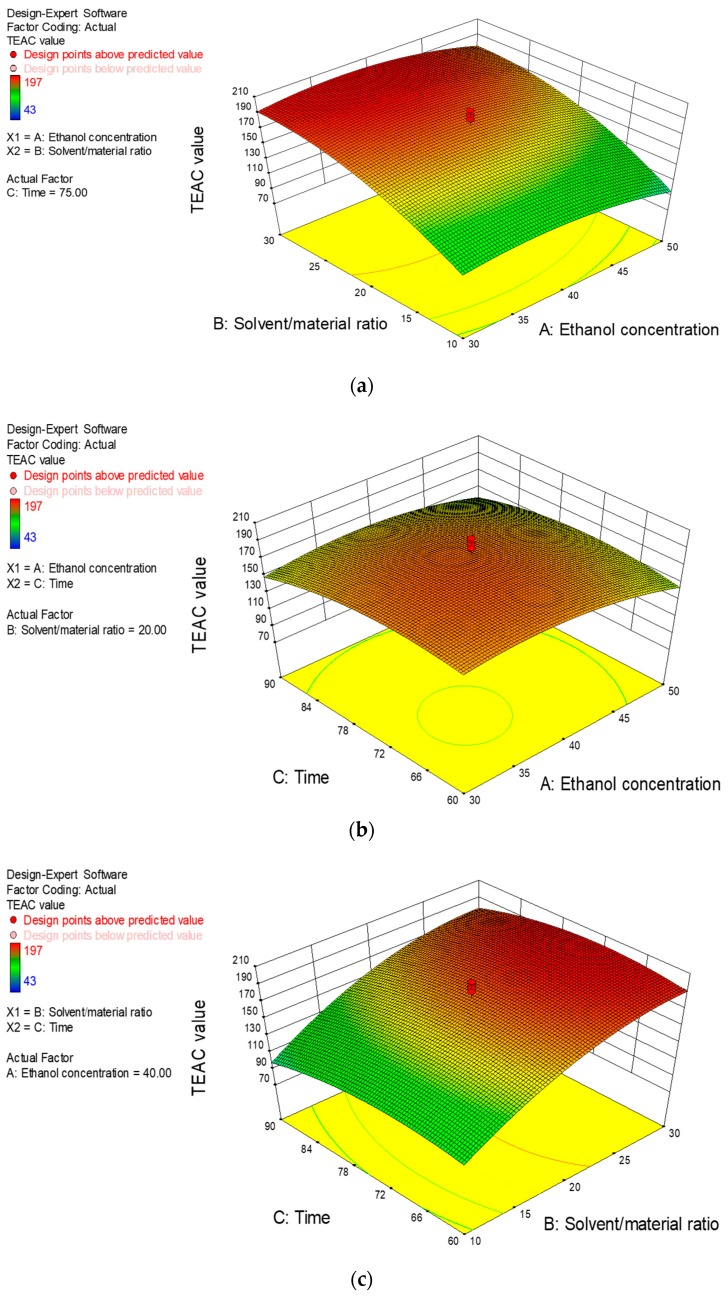
Response surface plots of the effects of ethanol concentration (%) and solvent/material ratio (mL/g) (**a**); ethanol concentration and extraction time (min) (**b**); and solvent/material ratio and extraction time (**c**) on TEAC value (μmol Trolox/g DW).

The three-dimensional response surfaces plots (Figure 3) illustrate the relationships between the response value (Y, TEAC value) and independent variables (X_1_, concentration of ethanol; X_2_, solvent/material ratio and X_3_, extraction time). According to Figure 3a, when the extraction time was set at 75 min, the response value elevated with the increasing ethanol concentration, but when the ethanol concentration exceeded 40%, the response value began to decrease. Besides, an increase of solvent/material ratio (from 10 to 30 mL/g) resulted in an increase of response value to the maximum. The S/M ratio exerted a strong influence on the response value, while the ethanol concentration caused a slighter influence. Figure 3b shows the effect of the interaction between ethanol concentration and extraction time on the response value when the S/M ratio was fixed at 20 mL/g. The increase of extraction time resulted in an initial increase of the response value and then a decrease as the time continued to increase. Meanwhile, it could be observed that the ethanol concentration exerted a similar influence on the TEAC values as that seen in Figure 3a. Figure 3c illustrates the interaction between solvent/material ratio and extraction time on the TEAC value at a fixed ethanol concentration of 40%. The solvent/material ratio and extraction time showed similar effects to those in Figure 3a,b. Combining the results of ANOVA in Table 2 and the response surfaces plots, conclusion could be drawn that solvent/material ratio (*p* < 0.01) exerted more significant influence on the response value than ethanol concentration and extraction time.

#### 2.2.4. Verification of Predicted Value

The optimal extraction conditions were obtained from the analysis of the quadratic polynomial regression model, and were as follows, ethanol concentration of 36.89%, S/M ratio of 29.56 mL/g, extraction time of 71.04 min, temperature of 40 °C, and microwave power of 400 W. The antioxidant activity of the extract was predicted as 200.28 µmol Trolox/g DW under the optimal conditions. Verification experiments under the abovementioned conditions (six replicates) were carried out to confirm the accuracy of the model. The result of TEAC value was 198.16 ± 5.47 µmol Trolox/g DW, which was in good agreement with the predicted value. 

### 2.3. Comparison of MAE with Conventional Extraction Methods

The efficacy of MAE was compared with those of conventional extraction methods (Soxhlet extraction and maceration), and the results are displayed in Table 3. The MAE process improved the extraction efficacy by 17% compared with maceration extraction. Furthermore, the MAE process significantly reduced the extraction time than the maceration method (71.04 min vs. 24 h). In comparison with Soxhlet extraction, the MAE process significantly increased the extraction efficacy by 74%, and meanwhile required lower temperature (40 °C vs. 85 °C) and shorter time (71.04 min vs. 4 h). The underlying mechanism of MAE is that microwave energy could cause molecular motion via ionic conduction and dipole rotation [49]. The resistance of the solution to this electrophoretic migration of ions would eventually cause rapid heating of the solution. Then the sudden increase in temperature could cause rupture of cell structures and promote the release of components in the cells. Besides, the rupture of cell structure is also reinforced by the cavitation and turbulence effect induced by microwave power, thus the mass transfer is further improved [47]. This can explain the high efficency of MAE. A similar result was reported in the study on extraction of triterpene acids from olive skins [50], and phenolic compounds from blueberry leaves [51]. Additionally, the extract obtained by MAE method showed higher total phenolic content (TPC) and total flavonoid content (TFC) than the other two extraction methods, which further demonstrated the high efficiency of MAE.

### 2.4. Analysis of Phenolic Components

**Figure 4:**
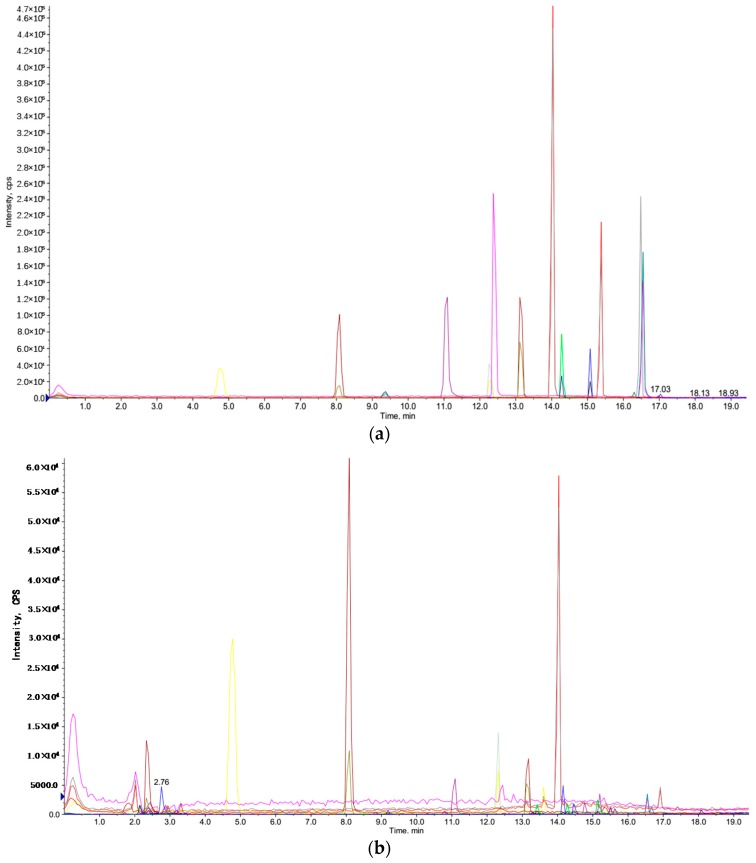
The total ion chromatograms of standard compounds (**a**) and the sample obtained under the optimal conditions (**b**).

Phenolic components exist extensively in fruits, and possess strong antioxidant activity [8,52,53]. Therefore, the identification of phenolic components in the extract from the fruit of *Gordonia axillaris* could help understanding its strong antioxidant capacity. In this study, ultra performance liquid chromatography-tandem mass spectrometry (UPLC-MS/MS) was employed to characterize the phenolic profiles in the fruit of *Gordonia axillaris*. The total ion chromatograms of standard compounds and the sample obtained under the optimal conditions are shown in Figure 4, and the lines with different colors are corresponded to different phenolic compounds. Ten phenolic components were identified (Table 4). The rutin showed the highest contents (60.38 ± 4.32 µg/g DW), followed by gallic acid, protocatechuic acid, epicatechin, epicatechin gallate, 2-hydrocinnamic acid, *p*-coumaric acid, quercetin, chlorogenic acid and ferulic acid. The strong antioxidant capacity of the *Gordonia axillaris* fruit might be attributed to the synergistic effect of these phenolic components, as well as other antioxidants. Besides, except for antioxidant capacity, these phenolic compounds have also shown other bioactivities, such as anticancer, anti-inflammatory and antibacterial activities [54,55,56,57,58,59], which indicated that the *Gordonia axillaris* fruit might possess these bioactivities. 

## 3. Materials and Methods 

### 3.1. Chemicals and Reagents

Trolox (6-hydroxy-2,5,7,8-tetramethylchromane-2-carboxylic acid), ABTS (2,2′-azinobis(3-ethyl-benothiazoline-6-sulphonic acid) diammonium salt), Folin-Ciocalteu’s phenol reagent, and phenolic compound standards (rutin, 2-hydrocinnamic acid, daidzein, equol, epigallocatechin, *p*-coumaric acid, glycitein, resveratrol, chlorogenic acid, quercetin, epicatechin, gallic acid, coffeic acid, epicatechin gallate, genistein, ferulic acid, and protocatechuic acid) were purchased from Sigma-Aldrich (St. Louis, MO, USA). Sodium carbonate, potassium persulphate, potassium acetate, and aluminum chloride hexahydrate were purchased from Tianjin Chemical Factory (Tianjin, China). Ethanol of analytical grade was used during the extraction process, and the formic acid and methanol of chromatographically pure grade were used in the UPLC-MS/MS analysis. All the other chemicals and reagents applied in this study were analytically pure, and deionized water was used.

### 3.2. Instrument

The MAE process was conducted in an X-100A microwave extraction device with a microwave power of 1000 W (Xianghu Instrumental Company, Beijing, China). The microwave extraction device was also equipped with a temperature monitor and a microprocessor programmer software to control the operation parameters, such as the temperature, running time and microwave power.

### 3.3. Sample Preparation

Mature fruits of *Gordonia axillaris* were harvested from different trees in the Lung Fu Mountain, Hong Kong. The fruits were washed with deionized water, air dried at room temperature, and then ground into fine particles by a special pulverizer (model XT-A400, Hongtaiyang Co., Ltd., Yongkang, Zhejiang, China) and sieved (0.300 mm particle size), and were stored at 4 °C in a refrigerator until further use.

### 3.4. Extraction and Evaluation of Natural Antioxidants

#### 3.4.1. Microwave-Assisted Extraction

The ground powder of *Gordonia axillaris* fruit (0.500 g) was put in a tube and mixed with ethanol aqueous solution (the concentration (*v*/*v*) and volume was decided according to the experimental design). After a 30 min of soaking which helped the solvent wet the material, the tube containing the mixture was immersed into a water bath placed in the microwave device, and irradiated under the pre-set conditions, including temperature, time and microwave power. After extraction, the mixture was centrifuged (4200× *g* for 15 min), and then the supernatant was gathered for the subsequent TEAC assay. Additionally, the supernatant was filtered using a 0.45 µm membrane for the subsequent UPLC-MS/MS analysis.

#### 3.4.2. Maceration Extraction

The ground sample (0.500 g) was placed in a centrifuge tube and mixed with 36.89% ethanol (14.78 mL), and extracted for 24 h at 25 °C in a shaking water bath. After the extraction, the mixture was centrifuged (4200× *g* for 15 min), and the supernatant was gathered for further analysis.

#### 3.4.3. Soxhlet Extraction

The Soxhlet extraction was conducted according to the method reported by Xu et al. [60]. The ground powder (0.500 g) of *Gordonia axillaris* fruit was wrapped with Whatman filter paper, and placed into a Soxhlet extractor. Then ethanol aqueous solution (36.89%, 400 mL) was added to the extractor. After 4 h of extraction under 95 °C (maintained by a water bath), the obtained solution was gathered for further analysis. 

#### 3.4.4. Determination of Antioxidant Capacity

The Trolox equivalent antioxidant capacity (TEAC) assay is a widely applied method for determination of the antioxidant activity of substances due to its efficiency, sensitivity and wide linear reaction range [18]. The results obtained from TEAC assay also often have a satisfactory correlation with the results from other methods such as FRAP assay [5,10]. Therefore, in this study, the antioxidant capacity of the extract was determined using TEAC assay based on the previously established procedure [5]. Briefly, a stock solution of ABTS^•+^ was prepared by mixing ABTS (7 mmol/L) and potassium persulfate (2.45 mmol/L) in a volume ratio of 1:1, and was then incubated for 16 h in dark at room temperature. The stock solution was used within 2 days. Then the working solution of ABTS^•+^ was obtained by diluting the ABTS^•+^ stock solution to ensure the absorbance reached 0.70 ± 0.05 at 734 nm. Finally, 3.8 mL of ABTS^•+^ working solution was mixed with a 100 µL of the dilute sample, and the mixture was incubated for 6 min at room temperature. After incubation, the absorbance was measured at 734 nm. The result of TEAC value was expressed as µmol Trolox/g DW.

#### 3.4.5. Determination of Total Phenolic Content

The total phenolic content (TPC) in the extract was assessed based on the method reported in the previous literature [11]. The gallic acid was used as the reference standard, thus the result of TPC was displayed as mg gallic acid equivalent (GAE)/g.

#### 3.4.6. Determination of Total Flavonoid Content

The total flavonoid content (TFC) in the extract was evaluated according to the method reported in the previous literature [61]. The quercetin was used as the reference standard, thus the result of TFC was displayed as mg quercetin equivalent (mg QE)/g DW.

#### 3.4.7. Analysis of Phenolic Components 

The phenolic components in the extract obtained under optimal condition was analyzed according to the method described previously by Zhou et al. with slight modifications [62]. An AB SCIEX 4000 QTRAP LC-MS/MS system (SCIEX, Framingham, MA, USA) was used, equipped with an Acquity UPLC^®^ HSS T3 column (3.0 × 150 mm, 1.8 µm, Waters, Milford, MA, USA) for the separation of phenolic components at 40 °C. The injection volume was 2 µL. The mobile phased consisted of solution A (0.2% formic acid aqueous solution) and solution B (methanol) with a flow rate of 0.3 mL/min. The gradient elution was programmed as follows: 0–2 min, 15% (B); 2–8 min, 15–30% (B); 8–15 min, 30–80% (B); 15–17.5 min, 80% (B); 17.5–19.5 min, 15% (B). For the conditions of MS, ESI source with negative mode was used and ion source temperature was 550 °C. Multiple reaction monitoring (MRM) mode was adopted and capillary voltage was −4500 V. The curtain gas, nebulizer gas and auxiliary gas was 12, 20, 20 psig, respectively. The phenolic compounds in the extracts were tentatively identified by MS/MS, then further verified and quantified by comparing the retention times and peak areas with those of standards (rutin, 2-hydrocinnamic acid, daidzein, equol, epigallocatechin, *p*-coumaric acid, glycitein, resveratrol, chlorogenic acid, quercetin, epicatechin, gallic acid, coffeic acid, epicatechin gallate, genistein, ferulic acid, and protocatechuic acid). The results were expressed as µg/g dry weight of *Gordonia axillaris* fruit.

### 3.5. Experimental Design

#### 3.5.1. Single-Factor Experiments

Single-factor experiments were conducted to evaluate the effects of five factors on the yield of natural antioxidant from the fruit of *Gordonia axillaris*, including ethanol concentration (20%, 30%, 40%, 50%, 60%, 70%, 80%), solvent/material ratio (10, 20, 30, 40, 50, 60 mL/g), extraction time (0, 15, 30, 45, 60, 75, 90 min), extraction temperature (30, 40, 50, 60, 70 °C), and microwave power (300, 400, 500, 600, 700, 800 W). After the single-factor experiments, three major influencing factors obtained would be selected for the following response surface method design. 

#### 3.5.2. Response Surface Methodology

By using a central composite rotatable design (CCRD), the RSM was carried out to optimize the yield of antioxidants extracted from the *Gordonia axillaris* fruit. According to the results from single-factor experiments, three independent variables, ethanol concentration (X_1_, %), S/M ratio (X_2_, mL/g), and extraction time (X_3_, min) were chosen for the RSM design. The independent variables and their related codes and levels are displayed in Table 5. A three-factor, five-level CCRD with 20 experimental runs was carried out, which included six replicates in the central point (Table 2). The response value of the 20 different runs was fitted to a second-order polynomial equation as follows:

Y = β_0_ + ∑β_i_X_i_ + ∑β_ii_X_i_^2^ + ∑β_ij_X_i_X_j_ (2)

### 3.6. Statistical Analysis

All the experiments in this study were performed in triplicate, and the results were expressed as mean value ± SD (standard deviation). The statistical analysis was carried out using Design Expert (version 8.0.6, Stat-Ease, Minneapolis, MN, USA) and Excel 2016 (Microsoft, Redmond, WA, USA). 

## 4. Conclusions

A microwave-assisted extraction method coupled with RSM has been developed in the present study to extract antioxidants from the fruit of *Gordonia axillaris* and to optimize the extraction conditions. A quadratic polynomial regression model was obtained, and the optimal extraction conditions obtained as follows, ethanol concentration of 36.89%, solvent/material ratio of 29.56 mL/g, extraction time of 71.04 min, extraction temperature of 40 °C, and microwave power of 400 W, and the TEAC value was 198.16 ± 5.47 µmol Trolox/g DW under the optimal conditions. The high R^2^ of 0.9183 as well as the consistency between the actual value and predicted value indicated the accuracy and reliability of the model. In addition, the antioxidant capacity as well as the total phenolic and flavonoid contents of the extract obtained by MAE method were higher than that obtained by maceration or Soxhlet extraction. Finally, several antioxidant components, including rutin, gallic acid, protocatechuic acid, epicatechin, epicatechin gallate, 2-hydrocinnamic acid, *p*-coumaric acid, quercetin, chlorogenic acid and ferulic acid, were identified in the extract and quantified. These phenolic components might contribute to the strong antioxidant capacity of *Gordonia axillaris* fruit. The present study proved that microwave-assisted extraction is indeed superior to the conventional extraction methods in some respects such as reducing extraction time, lowering extraction temperature, and reducing the use of organic solvents. In the future, the active components in the extract should be isolated and identified, and their antimicrobial and anticancer activities should be evaluated. Furthermore, the mechanisms of action should be studied.

## Figures and Tables

**Table 1:** The experimental design, experimental value, and predicted value of RSM.

Run	X_1_ (Ethanol Concentration, %)	X_2_ (Solvent/Material Ratio, mL/g)	X_3_ (Extraction Time, min)	Y (TEAC Value, µmol Trolox/g DW)	
				Actual Value	Predicted Value
1	56.82	20.00	75.00	110.06	124.18
2	40.00	20.00	75.00	196.88	179.29
3	30.00	10.00	90.00	95.46	90.30
4	50.00	10.00	90.00	94.47	77.98
5	40.00	20.00	75.00	150.63	179.29
6	40.00	20.00	75.00	162.84	179.29
7	40.00	36.82	75.00	183.81	187.90
8	50.00	30.00	60.00	171.90	165.03
9	40.00	20.00	100.23	113.47	128.99
10	40.00	20.00	49.77	150.92	152.42
11	30.00	30.00	90.00	176.86	168.81
12	50.00	30.00	90.00	165.73	159.57
13	30.00	30.00	60.00	183.28	187.73
14	30.00	10.00	60.00	118.59	112.71
15	40.00	20.00	75.00	192.67	179.29
16	23.18	20.00	75.00	150.72	153.63
17	50.00	10.00	60.00	90.91	86.93
18	40.00	3.18	75.00	43.27	56.21
19	40.00	20.00	75.00	185.82	179.29
20	40.00	20.00	75.00	189.81	179.29

**Table 2:** ANOVA of the fitted polynomial quadratic model.

Source	Sum of Squares	df	Mean Square	*F* Value	*p* Value	Significant
Model	32,462.64	9	3606.96	12.49	0.0002	significant
X_1_ (Ethanol concentration)	1046.75	1	1046.75	3.63	0.0861	
X_2_ (Solvent/material ratio)	20,933.85	1	20,933.85	72.50	<0.0001	
X_3_ (Time)	662.86	1	662.86	2.30	0.1607	
X_1_X_2_	4.75	1	4.75	0.016	0.9005	
X_1_X_3_	90.70	1	90.70	0.31	0.5875	
X_2_X_3_	6.09	1	6.09	0.021	0.8874	
X_1_^2^	2938.09	1	2938.09	10.18	0.0097	
X_2_^2^	5901.36	1	5901.36	20.44	0.0011	
X_3_^2^	2681.88	1	2681.88	9.29	0.0123	
Residual	2887.38	10	288.74			
Lack of Fit	1155.11	5	231.02	0.67	0.6663	not significant
Pure Error	1732.27	5	346.45			
Cor Total	35,350.01	19				
R-Squared	0.9183					
Adj R-Squared	0.8448					

**Table 3:** The comparison of MAE with conventional methods.

Extraction Methods	Ethanol Concentration (%)	Time	Temperature (°C)	TEAC (µmol Trolox/g DW)	TPC (mg GAE/g DW)	TFC (mg QE/g DW)
maceration	36.89	24 h	25	168.67 ± 3.88	13.69 ± 0.11	1.90 ± 0.08
Soxhlet	36.89	4 h	85	114.09 ± 2.01	9.63 ± 0.45	1.82 ± 0.11
MAE	36.89	71.04 min	40	198.16 ± 5.47	17.69 ± 1.02	3.11 ± 0.12

**Table 4:** The contents of phenolic components in extract obtained under the optimal condition.

Phenolic Components	Retention Time (*t*_R_, min)	Parent Ion (*m*/*z*, [M − H]¯)	Product Ion (*m*/*z*)	Contents (µg/g DW)
Rutin	14.77	609	300, 343	60.38 ± 4.32
Gallic acid	4.76	169.1	125, 112	15.39 ± 1.73
Protocatechuic acid	8.07	153.1	109, 108	11.03 ± 1.24
Epicatechin	12.3	289	203, 245	5.34 ± 0.44
Epicatechin gallate	13.13	441	169, 289.1	2.86 ± 0.31
2-Hydrocinnamic acid	14.03	163.1	119, 90	2.16 ± 0.23
*p*-Coumaric acid	14.03	162.7	119, 90	2.09 ± 0.21
Quercetin	16.54	301	179, 151	0.96 ± 0.05
Chlorogenic acid	11.07	353	191, 114	0.29 ± 0.02
Ferulic acid	14.28	193.1	134, 178	0.13 ± 0.01

**Table 5:** Five levels of the three variables of the extraction process.

Variable	Units	Symbol	Coded Levels				
			−1.68	−1	0	1	1.68
Ethanol concentration	% (*v*/*v*)	X_1_	23.18	30	40	50	56.82
Solvent/material ratio	mL/g	X_2_	3.18	20	30	40	36.82
Extraction time	min	X_3_	49.77	60	75	90	100.23

## References

[1] Lu J.M., Lin P.H., Yao Q.Z., Chen C.Y. (2010). Chemical and molecular mechanisms of antioxidants: Experimental approaches and model systems. J. Cell. Mol. Med..

[2] Halliwell B. (2011). Free radicals and antioxidants—Quo vadis?. Trends Pharmacol. Sci..

[3] Vina J., Gomez-Cabrera M.C., Lloret A., Marquez R., Minana J.B., Pallardo F.V., Sastre J. (2000). Free radicals in exhaustive physical exercise: Mechanism of production, and protection by antioxidants. IUBMB Life.

[4] Halliwell B. (2001). Role of free radicals in the neurodegenerative diseases-Therapeutic implications for antioxidant treatment. Drugs Aging.

[5] Fu L., Xu B.T., Xu X.R., Qin X.S., Gan R.Y., Li H.B. (2010). Antioxidant capacities and total phenolic contents of 56 wild fruits from South China. Molecules.

[6] Song F.L., Gan R.Y., Zhang Y.A., Xiao Q., Kuang L., Li H.B. (2010). Total phenolic contents and antioxidant capacities of selected Chinese medicinal plants. Int. J. Mol. Sci..

[7] Fu L., Xu B.T., Gan R.Y., Zhang Y.A., Xu X.R., Xia E.Q., Li H.B. (2011). Total phenolic contents and antioxidant capacities of herbal and tea infusions. Int. J. Mol. Sci..

[8] Fu L., Xu B.T., Xu X.R., Gan R.Y., Zhang Y., Xia E.Q., Li H.B. (2011). Antioxidant capacities and total phenolic contents of 62 fruits. Food Chem..

[9] Deng G.F., Xu X.R., Zhang Y., Li D., Gan R.Y., Li H.B. (2013). Phenolic compounds and bioactivities of pigmented rice. Crit. Rev. Food Sci..

[10] Deng G.F., Lin X., Xu X.R., Gao L.L., Xie J.F., Li H.B. (2013). Antioxidant capacities and total phenolic contents of 56 vegetables. J. Funct. Foods.

[11] Li A.N., Li S., Li H.B., Xu D.P., Xu X.R., Chen F. (2014). Total phenolic contents and antioxidant capacities of 51 edible and wild flowers. J. Funct. Foods.

[12] Gan R.Y., Shah N.P., Wang M.F., Lui W.Y., Corke H. (2016). Fermentation alters antioxidant capacity and polyphenol distribution in selected edible legumes. Int. J. Food Sci. Technol..

[13] Li Y., Zhang J.J., Xu D.P., Zhou T., Zhou Y., Li S., Li H.B. (2016). Bioactivities and health benefits of wild fruits. Int. J. Mol. Sci..

[14] Hsieh H.J., Fu C.H., Shih H.H. (2008). *Muribasidiospora gordoniae* sp. nov occurring on *Gordonia axillaris* in Taiwan. Bot. Stud..

[15] Chang C.W., Yang L.L., Yen K.Y., Hatano T., Yoshida T., Okuda T. (1994). Tannins from Theaceous plants. VII. new. Gamma-pyrone glucoside, and dimeric ellagitannins from *Gordonia axillaris*. Chem. Pharm. Bull..

[16] Power M., de Thompson M.C., Heese H.V., Louw H.H., Khan M.B. (1991). Priorities for provision of health care services for children in the Cape Province. S. Afr. Med. J..

[17] Wang C.C., Chen L.G., Yang L.L. (2001). Camelliin B induced apoptosis in HeLa cell line. Toxicology.

[18] Xu D.P., Li Y., Meng X., Zhou T., Zhou Y., Zheng J., Zhang J.J., Li H.B. (2017). Natural antioxidants in foods and medicinal plants: Extraction, assessment and resources. Int. J. Mol. Sci..

[19] Zhou Y., Zheng J., Gan R., Zhou T., Xu D., Li H. (2017). Optimization of ultrasound-assisted extraction of antioxidants from the mung bean coat. Molecules.

[20] Xu D.P., Zheng J., Zhou Y., Li Y., Li S., Li H.B. (2017). Ultrasound-assisted extraction of natural antioxidants from the flower of *Limonium sinuatum*: Optimization and comparison with conventional methods. Food Chem..

[21] Li A.N., Li S., Li Y., Xu D.P., Li H.B. (2016). Optimization of ultrasound-assisted extraction of natural antioxidants from the *Osmanthus fragrans* flower. Molecules.

[22] Wang H., Ding J., Ren N.Q. (2016). Recent advances in microwave-assisted extraction of trace organic pollutants from food and environmental samples. TrAC-Trend Anal. Chem..

[23] Sahin S., Samli R., Tan A., Barba F.J., Chemat F., Cravotto G., Lorenzo J.M. (2017). Solvent-free microwave-assisted extraction of polyphenols from olive tree leaves: Antioxidant and antimicrobial properties. Molecules.

[24] Perino-Issartier S., Abert-Vian M., Chemat F. (2011). Solvent free microwave-assisted extraction of antioxidants from sea buckthorn (*Hippophae rhamnoides*) food by-products. Food Bioprocess Technol..

[25] Mihiretu G.T., Brodin M., Chimphango A.F., Oyaas K., Hoff B.H., Gorgens J.F. (2017). Single-step microwave-assisted hot water extraction of hemicelluloses from selected lignocellulosic materials—A biorefinery approach. Bioresour. Technol..

[26] Florez N., Conde E., Dominguez H. (2015). Microwave assisted water extraction of plant compounds. J. Chem. Technol. Biot..

[27] Fang X.S., Wang J.H., Zhou H.Y., Jiang X.K., Zhu L.X., Gao X. (2009). Microwave-assisted extraction with water for fast extraction and simultaneous RP-HPLC determination of phenolic acids in Radix Salviae Miltiorrhizae. J. Sep. Sci..

[28] Jiang W.H., Shan H., Song J.Y., Lu H.T. (2017). Separation and purification of ombuoside from *Gynostemma pentaphyllum* by microwave-assisted extraction coupled with high-speed counter-current chromatography. J. Chromatogr. Sci..

[29] Xie X.J., Zhu D., Zhang W., Huai W.B., Wang K., Huang X.W., Zhou L., Fan H.J. (2017). Microwave-assisted aqueous two-phase extraction coupled with high performance liquid chromatography for simultaneous extraction and determination of four flavonoids in *Crotalaria sessiliflora* L.. Ind. Crop Prod..

[30] Katekhaye S.D., Laddha K.S. (2016). Microwave-assisted extraction and RP-HPLC quantification of bergapten from *Pithecellobium dulce*. Indian J. Pharm. Sci..

[31] Xia E.Q., Cui B., Xu X.R., Song Y., Ai X.X., Li H.B. (2011). Microwave-assisted extraction of oxymatrine from *Sophora flavescens*. Molecules.

[32] Xia E.Q., Wang B.W., Xu X.R., Zhu L., Song Y., Li H.B. (2011). Microwave-assisted extraction of oleanolic acid and ursolic acid from *Ligustrum lucidum* Ait. Int. J. Mol. Sci..

[33] Kala H.K., Mehta R., Sen K.K., Tandey R., Mandal V. (2016). Critical analysis of research trends and issues in microwave assisted extraction of phenolics: Have we really done enough. TrAC-Trend. Anal. Chem..

[34] Ameer K., Bae S.W., Jo Y., Lee H.G., Ameer A., Kwon J.H. (2017). Optimization of microwave-assisted extraction of total extract, stevioside and rebaudioside-A from *Stevia rebaudiana* (Bertoni) leaves, using response surface methodology (RSM) and artificial neural network (ANN) modelling. Food Chem..

[35] Sinha K., Chowdhury S., Das Saha P., Datta S. (2013). Modeling of microwave-assisted extraction of natural dye from seeds of *Bixa orellana* (Annatto) using response surface methodology (RSM) and artificial neural network (ANN). Ind. Crop Prod..

[36] Lefsih K., Giacomazza D., Dahmoune F., Mangione M.R., Bulone D., Biagio P., Passantino R., Costa M.A., Guarrasi V., Madani K. (2017). Pectin from *Opuntia ficus* indica: Optimization of microwave-assisted extraction and preliminary characterization. Food Chem..

[37] Yanik D.K. (2017). Alternative to traditional olive pomace oil extraction systems: Microwave-assisted solvent extraction of oil from wet olive pomace. LWT-Food Sci. Technol..

[38] Bhan M., Satija S., Garg C., Dureja H., Garg M. (2017). Optimization of ionic liquid-based microwave assisted extraction of a diterpenoid lactone-andrographolide from *Andrographis paniculata* by response surface methodology. J. Mol. Liq..

[39] Chen C., Shao Y., Tao Y.D., Wen H.X. (2015). Optimization of dynamic microwave-assisted extraction of Armillaria polysaccharides using RSM, and their biological activity. LWT-Food Sci. Technol..

[40] Moreira M.M., Barrosoa M.F., Boeykens A., Withouck H., Morais S., Delerue-Matos C. (2017). Valorization of apple tree wood residues by polyphenols extraction: Comparison between conventional and microwave-assisted extraction. Ind. Crop Prod..

[41] Dailey A., Vuong Q. (2015). Optimization of aqueous extraction conditions for recovery of phenolic content and antioxidant properties from Macadamia (*Macadamia tetraphylla*) skin waste. Antioxidants.

[42] Cheng Z.Y., Song H.Y., Cao X.L., Shen Q.H., Han D.D., Zhong F.L., Hu H.B., Yang Y.J. (2017). Simultaneous extraction and purification of polysaccharides from *Gentiana scabra* Bunge by microwave-assisted ethanol-salt aqueous two-phase system. Ind. Crop Prod..

[43] Varadharajan V., Shanmugam S., Ramaswamy A. (2017). Model generation and process optimization of microwave-assisted aqueous extraction of anthocyanins from grape juice waste. J. Food Process Eng..

[44] Chen C., Zhang B., Huang Q., Fu X., Liu R.H. (2017). Microwave-assisted extraction of polysaccharides from *Moringa oleifera* Lam. leaves: Characterization and hypoglycemic activity. Ind. Crop Prod..

[45] Tang X.Y., Zhu D., Huai W.B., Zhang W., Fu C.J., Xie X.J., Quan S.S., Fan H.J. (2017). Simultaneous extraction and separation of flavonoids and alkaloids from *Crotalaria sessiliflora* L. by microwave-assisted cloud-point extraction. Sep. Purif. Technol..

[46] Ghasemzadeh A., Jaafar H., Rahmat A., Swamy M.K. (2017). Optimization of microwave-assisted extraction of zerumbone from *Zingiber zerumbet* L. rhizome and evaluation of antiproliferative activity of optimized extracts. Chem. Cent. J..

[47] Hu B., Li C., Zhang Z.Q., Zhao Q., Zhu Y.D., Su Z., Chen Y.Z. (2017). Microwave-assisted extraction of silkworm pupal oil and evaluation of its fatty acid composition, physicochemical properties and antioxidant activities. Food Chem..

[48] Li Q., Fu C. (2005). Application of response surface methodology for extraction optimization of germinant pumpkin seeds protein. Food Chem..

[49] Teo C.C., Chong W., Ho Y.S. (2013). Development and application of microwave-assisted extraction technique in biological sample preparation for small molecule analysis. Metabolomics.

[50] Fernandez-Pastor I., Fernandez-Hernandez A., Perez-Criado S., Rivas F., Martinez A., Garcia-Granados A., Parra A. (2017). Microwave-assisted extraction versus Soxhlet extraction to determine triterpene acids in olive skins. J. Sep. Sci..

[51] Routray W., Orsat V. (2014). MAE of phenolic compounds from blueberry leaves and comparison with other extraction methods. Ind. Crop Prod..

[52] Deng G.F., Xu D.P., Li S., Li H.B. (2015). Optimization of ultrasound-assisted extraction of natural antioxidants from sugar apple (*Annona squamosa* L.) peel using response surface methodology. Molecules.

[53] Li F., Li S., Li H.B., Deng G.F., Ling W.H., Wu S., Xu X.R., Chen F. (2013). Antiproliferative activity of peels, pulps and seeds of 61 fruits. J. Funct. Foods.

[54] Liu Y., Tang Z.G., Yang J.Q., Zhou Y., Meng L.H., Wang H., Li C.L. (2017). Low concentration of quercetin antagonizes the invasion and angiogenesis of human glioblastoma U251 cells. OncoTargets Ther..

[55] Aglan H.A., Ahmed H.H., El-Toumy S.A., Mahmoud N.S. (2017). Gallic acid against hepatocellular carcinoma: An integrated scheme of the potential mechanisms of action from in vivo study. Tumor Biol..

[56] Zhang Z.H., Pan T.W. (2017). HPLC determination of chlorogenic acid in *Verbena officinalis* L. extract and its in vitro antibacterial activity. Biomed. Res.-India.

[57] Park W.H. (2017). Gallic acid induces HeLa cell death via increasing GSH depletion rather than ROS levels. Oncol. Rep..

[58] Khan A.K., Rashid R., Fatima N., Mahmood S., Mir S., Khan S., Jabeen N., Murtaza G. (2015). Pharmacological activities of protocatechuic acid. Acta Pol. Pharm..

[59] Habtemariam S., Lentini G. (2015). The therapeutic potential of rutin for diabetes: An update. Mini-Rev. Med. Chem..

[60] Xu D.P., Zheng J., Zhou Y., Li Y., Li S., Li H.B. (2016). Extraction of natural antioxidants from the *Thelephora ganbajun* mushroom by an ultrasound-assisted extraction technique and evaluation of antiproliferative activity of the extract against human cancer cells. Int. J. Mol. Sci..

[61] Kalia K., Sharma K., Singh H.P., Singh B. (2008). Effects of extraction methods on phenolic contents and antioxidant activity in aerial parts of *Potentilla atrosanguinea* Lodd. and quantification of its phenolic constituents by RP-HPLC?. J. Agric. Food Chem..

[62] Zhou T., Xu D.P., Lin S.J., Li Y., Zheng J., Zhou Y., Zhang J.J., Li H.B. (2017). Ultrasound-assisted extraction and identification of natural antioxidants from the fruit of *Melastoma sanguineum* Sims. Molecules.

